# Guanosine protects against glycerol-induced acute kidney injury via up-regulation of the *klotho* gene

**DOI:** 10.22038/ijbms.2022.60579.13428

**Published:** 2022-03

**Authors:** Rasha F. Ahmed, Ahmed M. Okasha, Salwa Hamdy Ibrahim Hafiz, Seham A. Abdel-Gaber, Rehab K. Mohamed Yousef, Wael F Sedik

**Affiliations:** 1 Department of Medical Biochemistry, Faculty of Medicine, Minia University, 61511, Minia, Egypt; 2 Department of Pharmacology, Faculty of Medicine, Minia University, 61511, Minia, Egypt; 3 Department of Pathology, Faculty of Medicine, Minia University, 61511, Minia, Egypt

**Keywords:** Acute kidney injury, Caspase-3, Glycerol, Guanosine, Klotho, Rhabdomyolysis

## Abstract

**Objective(s):**

Acute Kidney Injury (AKI) is characterized by a rapid and reversible decline in renal function with a rapid decrease in Glomerular Filtration Rate (GFR), which is associated with high mortality. Rhabdomyolysis accounts for 10–40% of AKI, to which the therapeutic approach is limited. *Klotho* is a protein that modulates sodium-phosphate co-transporters, ion channels that have been reported to have a renal protective effect. Guanosine, a purine nucleoside, has already been reported to have a renal protective effect; however, the mechanism of such protection and its relation to *Klotho* modification has not been evaluated yet. This study aims to evaluate the mechanism of the protective effect of guanosine against rhabdomyolysis-induced AKI and its relation to the expression of the *Klotho* gene.

**Materials and Methods:**

In the current study, rats were divided into three groups: control, glycerol-induced AKI, and guanosine-treated. Serum urea and creatinine levels, renal tissue Total Antioxidant Capacity (TAC), and *Klotho* and *Cystatin C* genes expression were evaluated. Furthermore, caspase-3 immunostaining and histopathological evaluations were done.

**Results:**

Results showed that guanosine treatment resulted in a significant reduction in serum urea and creatinine, *Cystatin C *genes expression, and caspase-3 immunoexpression, and an increase in TAC and *Klotho* genes expression. Results also revealed an improvement of renal histopathology when compared with the glycerol-induced AKI group.

**Conclusion:**

Guanosine may be a promising agent in the treatment of rhabdomyolysis-induced AKI. The proposed mechanism for guanosine may be through its ability to enhance *Klotho* gene expression in renal tissue, with subsequent antioxidant and anti-apoptotic activity.

## Introduction

Rhabdomyolysis is a syndrome characterized by rapid dissolution of damaged or injured skeletal muscles, which causes myoglobin, other intracellular proteins, and electrolytes to leak into the circulation (1). 10–50% of patients with rhabdomyolysis develop Acute Kidney Injury (AKI) (2). The pathological mechanisms of rhabdomyolysis-induced AKI involve tubular obstruction, renal vasoconstriction, and oxidative stress (1). It was also reported that apoptosis of renal tubular epithelial cells is one of the main contributors to pathogenesis of this form of AKI (3). Activated caspase-3, which is one of the multiple biomarkers involved in apoptosis, is one of the family members of cysteine aspartate-directed protease. It is systematically activated from inactive zymogen-like states, and upon maturation, it targets a host of cellular proteins for hydrolysis. The induction of apoptosis leads to both protein substrate degradation and activation, followed by cell death (4). Glycerol is one of the most commonly used agents for experimental induction of AKI. It is characterized by inflammatory cellular infiltration and intense acute cortical tubular necrosis simulating that of rhabdomyolysis (5-7).

*Klotho* is a membrane-bound protein expressed mainly in the kidney, mostly in the distal tubule, and at a low level in the proximal tubule. It plays a critical role in renal protection by its antioxidant, anti-inflammatory, and anti-apoptotic effects. Its renal protective activity against rhabdomyolysis-induced AKI was confirmed (8, 9). Thus, modification and overexpression of the *Klotho* gene might be a good candidate for further therapy for AKI in clinical trials.

Guanosine is a purine nucleoside that is composed of guanine attached to a ribose via a β-N9-glycosidic bond. It can be phosphorylated to Guanosine Monophosphate (GMP), cyclic Guanosine Monophosphate (cGMP), Guanosine Diphosphate (GDP), and Guanosine Triphosphate (GTP) (10). These forms play important roles in various biochemical processes such as synthesis of nucleic acids and proteins, photosynthesis, muscle contraction, and intracellular signal transduction (11).

The protective effect of guanosine has been already evaluated in renal damage induced by ischemia-reperfusion; however, its role in rhabdomyolysis-induced AKI and its relation to *Klotho* gene modification has not been evaluated yet, to the researcher’s best knowledge. Therefore, the current study aims to evaluate the protective effect of guanosine against impaired renal function associated with rhabdomyolysis. The study also aims to assess the mechanism of the protection, if found, and its relation to *Klotho* gene expression.

## Materials and Methods

***Drugs, kits, and antibodies***

Glycerol and guanosine were procured from Amersham Biosciences AB (Sweden, EU) and Sigma-Aldrich (St Louis, MO, USA), respectively. Urea and creatinine colorimetric kits were from BioMED Diagnostic (Cairo, Egypt). The Total Antioxidant Capacity (TAC) assay kit was from Abcam, (Cambridge, MA, USA), caspase-3 rabbit polyclonal antibodies were from Thermo Fisher Scientific Inc./Lab Vision (Fremont, CA, USA). All other chemicals were for analytical purposes and were purchased from their commercial sources.

***Animals***

Thirty male Wistar rats (200-250 g) were used for this study. Animals were acclimatized for 7 days before starting the study. All animal care and procedures were in compliance with the National Institutes of Health Guidelines for the Care and Use of Laboratory Animals. Animals were housed in stainless steel cages and had access to food and water *ad libitum* during the study. Ethical approval was obtained for the study from the Research Ethics Committee, Faculty of Medicine, Minia University (approval number: 4152021).

***Grouping***

The animals were randomly allocated into three equal groups (n=10) as follows: Control group, in which rats were injected via single intramuscular (IM) injection of distilled water and daily intraperitoneal (IP) injection of sterile 0.9% NaCl solution for 2 weeks before distilled water injection, then completed for one month after injection to complete the 6 weeks until scarification. Glycerol induced AKI group, in which AKI was induced by a single injection of glycerol 50% v/v in distilled water in a dose of (8 ml/kg, IM) equally distributed to both hind legs and daily IP injection of sterile 0.9% NaCl solution for 2 weeks before distilled water injection, then completed for one month after AKI induction (12). Rats were allowed free access to food but deprived of drinking water for 24 hr before glycerol injection. The guanosine-treated group, in which guanosine was given 30 mg/kg/day, IP, dissolved in sterile 0.9% NaCl solution for 2 weeks before induction of AKI, then completed for one month after glycerol induction (10). 

***Collection of samples***

At the end of the experiment, blood samples were collected to estimate the serum levels of urea and creatinine. Kidney tissues were excised and divided into 3 parts; one was homogenized in 10% w/v ice-cold phosphate buffer (0.01 M, pH 7.4). The homogenate was centrifuged at 3000 rpm for 20 min and the supernatant was used for estimation of TAC. Another part was fixed in 10% formalin for histopathological study and immunohistochemistry. The last part of the kidney was stored at -80 ^°^C until used for RNA extraction. 

***Biochemical measurements***

*Estimation of serum urea and creatinine level*

Serum urea and creatinine were measured using commercial colorimetric kits according to kit instructions. The results were expressed as mg/dl.

*Estimation of renal TAC*

TAC was evaluated using a colorimetric kit according to the kit instructions, and the result was expressed as mM/g protein. Total protein was determined by the Coomassie binding method (13).

*Real-time reverse transcriptase polymerase chain reaction (RT-PCR)*

Total RNA was extracted from the renal tissue of experimental rats using Ribozol solution (Zymo Research, California, USA). RNA concentration was measured by a spectrophotometer (Nanodrop 2000, Thermo Scientific, USA) in which 260/280 more than 2 and 260/230 more than 1.8 were intact. 2 μg of total RNA of each specimen was converted into cDNA using RT First Strand kit (Qiagen Sciences, Maryland, USA). Gene expression was examined for *Klotho* and Cystatin C genes. β actin was included as an internal control and for normalization.

The primer used for *Cystatin C* gene was 5’ –TTCCAGCCACAAGCTGCTTA- ‘3 and 5’–AACAAGGGCAGCAACGAT-’3 (14) and for the *Klotho* gene; 5’-CGTGAATGAGGCTCTGAAAGC-3’ and 5’-CGTGAATGAGGCTCTGAAAGC-3 (15). The primer of *β actin* gene; 5’-CCCATTGAACACGGCATT G-3’ and 5’-GTACGACCAGAGGCATACA-3’ (15).

Amplifications were performed in 25 μl reaction volume in each tube, which contained 12.5 μl SYBR Green (SensiFast SYBR, Bioline, UK), 1 μl of cDNA template, 2 μl of 10 pM primers, and 9.5 μl of nuclease-free water. Cycling protocol of PCR amplification was done as follows: initial denaturation at 95 ^°^C for 2 min, followed by 40 cycles of amplification (denaturation at 94 ^°^C for 20 sec, annealing and extension at 60 ^°^C for 1 min). For each sample, the procedure was carried out in triplicate. Gene expression was expressed relative to that of the control group.

*Histopathological examination:*

Kidney samples obtained from all groups were fixed in 10% buffered formalin (pH 7.4 then dehydrated in ascending grades of alcohol and impregnated and embedded in paraffin at 55 ^°^–60 ^°^C, then 4 μm thick sections were cut and subjected to hematoxylin and eosin stain (16), then examined under a microscope to evaluate pathological changes by a pathologist who was blind to the data of the groups. 

***Immunohistochemical staining for caspase-3***

Sections (4 μm thick) of the kidney specimens were deparaffinized with xylene and rehydrated by using graded ethanol. 0.5% H_2_O_2_ was used for quenching endogenous peroxides then nonspecific binding was blocked by normal goat serum diluted 1:50 in 0.1M phosphate buffer. Antigen retrieval was done by immersion slides in citrate buffer (pH 6.0) using microwave. Afterward, slides were left to cool at room temperature and washed in PBS solution. Tissues were incubated in the primary antibody, polyclonal rabbit/anti-rat caspase-3 antibody (1:1000) overnight at 4 ^°^C. Afterward, tissues were washed then incubated in biotinylated goat anti-rabbit secondary antibody (Transduction Laboratories, USA) (1:2000) for 30 min, slides were then washed in PBS. The brownish color was developed by using 3,3-diaminobenzidinetetra hydrochloride (DAB). Slides were washed in distilled water, stained with hematoxylin, dehydrated, cleared by xylene, and covered. Immunohistochemically caspase-3 expression was confirmed by cytoplasmic and/ or nuclear staining in the examined cells.

***Immunohistochemical scoring of caspase-3***

For each specimen, caspase-3 immunoreactivities were examined in 10 randomly selected areas using x 100 objectives. Staining intensity was scored as 0 (negative), 1 (weak), 2 (moderate), and 3 (strong). Staining extent was scored as 0 (0%), 1 (1-25%), 2 (26-50%), 3 (26-75), and 4 (76-100). The sum of the intensity and extent of scores was calculated to estimate the final average staining scores (17).

***Statistical analysis***

Results were expressed as means±SEM. The statistical analysis was done using GraphPad Prism software version 6.01 (San Diego, CA, USA). One-way analysis of variance (ANOVA) followed by Tukey’s post-analysis test was used to analyze the results for a statistically significant difference. Differences with *P*-value<0.05 were considered significant.

## Results

***Serum urea and creatinine and renal tissue TAC in study groups***

Induction of AKI using glycerol resulted in a significant elevation of the serum urea and creatinine levels and reduction of TAC compared with the control group. Treatment by guanosine decreased both urea and creatinine levels and increased TAC in comparison with AKI rats (Table 1). 

***Relative quantitative expression of cystatin C and clotho genes***

**Figure 1:**
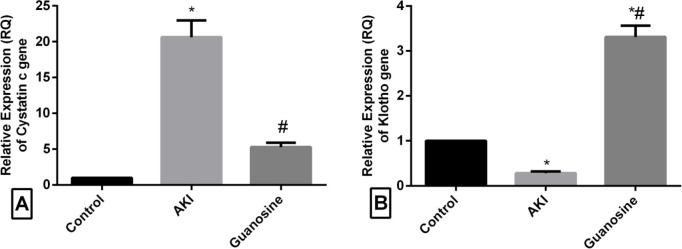
Effect of treatment with guanosine on the relative expression (RQ) of *cystatin C* and *Klotho* genes in glycerol induced AKI compared with the control group

The relative quantitative expression of the *Cystatin C* gene was significantly increased, however, the relative quantitative expression of the *Klotho* gene was significantly decreased in the glycerol-induced AKI group compared with the normal control group. On the other hand, guanosine treatment significantly decreased the relative expression of the *Cystatin C* gene and highly enhanced the *Klotho* gene expression compared with the glycerol-induced AKI group (Figure 1).

***Histopathological results***

**Figure 2:**
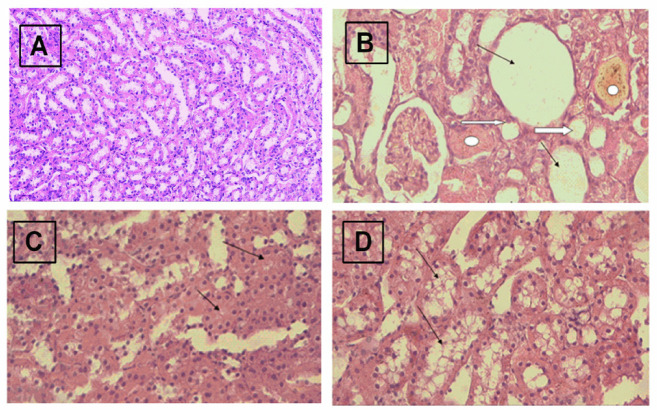
Photomicrographs of H&E-stained sections of kidney tissue (H&E, x20:40). (A) Control group showing no pathological abnormalities in the tubules. (B) Glycerol-induced AKI group showed a tubular lesion in the form of tubular dilatation (thin arrow) and tubular atrophy (thick arrow) granular cast (circle). (C) Cells showing cloudy swelling (arrow) (D) Guanosine treated group showed decreasing severity of cloudy swelling with reformation of the lumen and disappearance of the casts

The photomicrographs of H&E-stained sections of kidney tissue indicate the difference between the study groups. While no pathological abnormalities in the tubules were observed in the control group, tubular lesions in the form of tubular dilatation with flattened epithelium, tubular atrophy, and granular casts within the tubules were observed in glycerol induced the AKI group, also, there was an area showing cloudy swelling. The guanosine treated group showed a decrease in the severity of cloudy swelling with reformation of the lumen and disappearance of the casts (Figure 2).

***Immunohistochemical staining of caspase-3***

**Figure 3:**
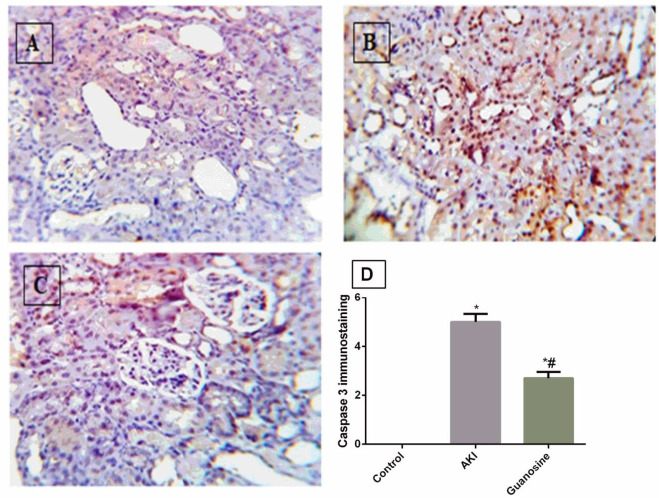
Immunohistochemical staining of caspase-3 in kidney tissues (x40). (A) Control group showed no immunoreactivity of caspase 3. (B) Glycerol induced AKI group showed increased immunoexpression of caspase-3 in the tubules. (C) Guanosine treated group showed reduced immunoreactivity of caspase 3 in the tubules. (D) Quantification of caspase-3optical density. Values represent the mean ± SEM. (n=10). *# significantly different (P<0.05) from control and AKI groups, respectively

Normal kidney tissue in the control group showed no immunoreactivity of caspase-3 (Figure 3A). While increased immunoexpression of caspase-3 in the renal tubules was observed in the glycerol-induced AKI group, which was quantitatively higher than the control rats in a significant manner (Figures 3B, D). The guanosine-treated group showed significantly decreased immunoexpression of caspase-3 in the renal tubules in comparison with the AKI group (Figures 3C, D).

**Table 1:** Effect of guanosine on the serum levels of urea and creatinine and renal tissue TAC in glycerol induced AKI

**Groups**	**Urea (mg/dl)**	**Creatinine (mg/dl)**	**TAC (nM/g protein)**
**Control**	31.10 ± 1.37	0.79 ± 0.06	4.22 ± 0.23
**AKI**	229.7 ± 20.26^*^	3.8 ± 0.34^*^	1.51 ± 0.15^*^
**Guanosine**	63.40 ± 5.84^#^	0.86 ± 0.07^#^	2.31 ± 0.04^*#^

Values represent the mean±SEM. (n=10). *#significantly different (*P<*0.05) from control and AKI groups, respectively

AKI: Acute Kidney Injury. TAC: Total Antioxidant Capacity

Values represent the mean±SEM. (n=10). *# significantly different (*P<*0.05) from control and AKI groups, respectively

AKI: Acute Kidney Injury

AKI: Acute Kidney Injury

## Discussion

AKI is a global public health problem characterized by a rapid renal function decline resulting in accumulation of nitrogenous waste substances in blood, which in turn increases serum urea and creatinine levels within hours to days (18). 

Rhabdomyolysis is a syndrome characterized by skeletal muscle degeneration and muscle enzyme leakage, which result in high mortality. Glycerol was commonly used for experimental induction of AKI. The effectiveness of glycerol in induction of acute renal failure was determined by serum creatinine and urea levels and by histopathological findings (19). Glycerol produces a myoglobinuric state similar to clinical rhabdomyolysis, characterized by rapid increases in blood urea and creatinine. Moreover, the heme of myoglobin induces oxidative stress and lipid peroxidation of the proximal tubular cells, triggering the release of a series of mediators, e.g., cytokines and chemokines. This causes leukocyte activation with subsequent tubular necrosis in the renal cortical area and a marked reduction in Glomerular Filtration Rate (GFR) (20, 21). 

In the current study, IM injection of glycerol resulted in renal damage, which is manifested by elevated serum levels of urea and creatinine compared with the control group. Moreover, the histopathological examination of the kidney tissues revealed tubular lesions in the form of tubular dilatation with hyaline casts, tubular atrophy, and cloudy swelling in the cells lining the tubules. Our findings are in accordance with the previously published studies (1, 22). On the contrary, treatment with guanosine normalized both urea and creatinine levels, decreased the severity of cloudy swelling of cells, and caused casts to disappear. In accordance with the current findings, Kelly and his colleagues, previously reported the renal protective activity of guanosine against ischemic injury in mice (10). 

In accordance with the previously published studies (23, 24), our study revealed induction of oxidative stress in the renal tissue by glycerol injection, which is manifested by decreased renal TAC in comparison with the normal control group. On the contrary, treatment with guanosine protected the renal tissue and restored the oxidative stress/antioxidant balance, which is confirmed by elevation of renal TAC in comparison with the AKI group. The antioxidant activity of guanosine may be one of the main contributory factors of its protection against glycerol-induced AKI; this activity was previously reported in studies conducted on various tissues. Gudkov and his colleagues (25) have reported that guanosine is a natural antioxidant that prevented oxidative damage to DNA, decreased generation of reactive oxygen species, and protected mice against gamma-radiation-induced death. Moreover, it was found that guanosine could modulate the methylmercury-induced oxidative stress in rat brain cortical slices in a simple* in vitro* model (26). 

Cystatin C is a nonglycosylated basic protein that is produced at a constant rate by all cells. It is freely filtered by the renal glomeruli but not secreted or reabsorbed as an intact molecule, as it is primarily catabolized in the tubules. Serum Cystatin C concentration is considered an improved marker of GFR, regardless of age, sex, and muscle mass (27). Cystatin C has an important role in regulation of the apoptosis caused by oxidative stress (28). In the current study, we found that glycerol administration significantly induced expression of the renal Cystatin C compared with the control group, indicating that *Cystatin C* gene expression is up-regulated in AKI. On the other hand, the expression of the *Cystatin C* gene was significantly decreased in the guanosine-treated group as compared with the glycerol-induced AKI group. This indicates that guanosine was able to down-regulate the expression of the *Cystatin C* gene in the kidney tissue that regulates apoptosis. This may be done by its antioxidant activity that was detected in this study. In line with the present findings, a study (28) reported that oxidative stress stimulated an increase in Cystatin C expression in cultured neurons. Moreover, researchers demonstrated that both oxidative stress and Cystatin C levels were increased in patients with preeclampsia. They also reported that the increased Cystatin C levels were secondary to oxidative stress (29).

*Klotho* gene is expressed in multiple tissues, with the kidney being the organ where it is primarily expressed, especially in the distal tubule, but also in the collecting duct and the proximal tubule lumen (30). Under normal physiological conditions, the kidney is a major regulator that helps in maintaining the *klotho* level; however, in chronic kidney disease, the *Klotho* level declines. This decline is accompanied by renal insufficiency (31). Experimental models have already reported that development and progression of kidney diseases were significantly associated with a decline in *Klotho* (32, 33). Moreover, agents enhancing *Klotho* gene expression showed renal-protective activity (8, 34, 35). 

The current study showed that the expression of the rat *Klotho* gene in the kidney tissues was significantly decreased in the glycerol-induced AKI group, compared with the control group. This confirmed that *Klotho* gene expression is down-regulated in acute renal failure. This result was in agreement with that of Sharifian *et al*. (36) who found that expression of the *Klotho* gene in the kidney was decreased in mice models of AKI. On the contrary, expression of the *Klotho* gene in renal tissue was significantly increased in the guanosine-treated group compared with the glycerol-induced group. This indicates that guanosine was able to up-regulate the expression of the *Klotho* gene in kidney tissues. It was reported that the activity of *Klotho* protein was mediated by cAMP and NO-cGMP-dependent pathway (37). Furthermore, *Klotho* pretreatment protected against cell death by decreasing the activity of caspase-9 and caspase-3 (38). This agreed with our results, which showed that guanosine up-regulated *Klotho* gene expression and decreased caspase-3 in renal tissues.

Caspases are the main stimulators of apoptosis. They participate in the pathogenic mechanisms in glycerol-induced AKI such as inflammation, apoptosis, vasoconstriction, and tubular necrosis. Early inhibition of caspases can attenuate these mechanisms and reduce renal function impairment (3).

The current study showed an increase of immunohistochemical staining of caspase-3 in kidney sections of the glycerol-treated group, with significant semi-quantitative scoring of the positive cells in comparison with the normal control group. On the other hand, treatment with guanosine decreased the caspase-3 immunostaining to a significant quantitative level when compared with the AKI non-treated group. This indicates a possible anti-apoptotic effect of guanosine. This observation is consistent with the previous study of Kelly *et al*. (10), who found that treatment with guanosine increased the GTP level and reduced medullary apoptosis after ischemia-reperfusion injury.

To sum up, guanosine treatment in glycerol-induced AKI resulted in improvement of the kidney function in the form of lowered serum levels of creatinine and urea, increased total renal antioxidant capacity, lower degree of renal tubular lesions, and down-regulation of *Cystatin C* gene expression. All these effects were associated with up-regulation of *Klotho* gene expression and decrease in caspase-3 immuno-expression.

## Conclusion

Based on the present findings, guanosine may be considered as a promising agent in the protection against or treatment of rhabdomyolysis-induced AKI because of its capability of inducing *Klotho* gene expression that may help in reducing apoptosis and oxidative stress and increasing renal TAC. However, further studies are required to investigate the effect of guanosine on the target protein level, to confirm our proposed hypothesis.

## Authors’ Contributions

RFA Supervised the research; AMO and SHI Prepared the draft manuscript; WFS and SAA Critically revised the paper. RKM did the histopathological and immunohistochemical part of the research; SAA and RFA Read and approved the final version to be published.

## Funding

This research did not receive any specific grant from funding agencies in the public, commercial, or not-for-profit sectors.

## Conflicts of Interest

The authors declare that they have no conflicts of interest.
